# Metabolic phenotype analysis of *Trichophyton rubrum* after laser irradiation

**DOI:** 10.1186/s12866-023-02759-3

**Published:** 2023-01-21

**Authors:** Ruina Zhang, Junying Zhao, Linfeng Li

**Affiliations:** Department of Dermatology, Beijing Friendship Hospital, Capital Medical University, 95 Yongan Road, Xicheng District, 100050 Beijing, China

**Keywords:** *Trichophyton rubrum*, Laser irradiation, Phenotype microarray system, Metabolic phenotype

## Abstract

**Background:**

Biological phenotypes are important characteristics of microorganisms, and often reflect their genotype and genotype changes. Traditionally, *Trichophyton rubrum* (*T. rubrum*) phenotypes were detected using carbon source assimilation tests, during which the types of tested substances are limited. In addition, the operation is complicated, and only one substance can be tested at once. To observe the changes of the metabolic phenotype of *T. rubrum* after laser irradiation, a high-throughput phenotype microarray system was used to analyze the metabolism of different carbon, nitrogen, phosphorus and sulfur source substrates in a Biolog metabolic phenotyping system.

**Results:**

The strain of *T. rubrum* used in this study can effectively utilize 33 carbon, 20 nitrogen, 16 phosphorus, and 13 sulfur source substrates prior to laser irradiation. After laser irradiation, the strain was able to utilize 10 carbon, 12 nitrogen, 12 phosphorus, and 8 sulfur source substrates. The degree of utilization was significantly decreased compared with the control. Both groups efficiently utilized saccharides and organic acids as carbon sources as well as some amino acids as nitrogen sources for growth. The number of substrates utilized by *T. rubrum* after laser irradiation were significantly reduced, especially carbon substrates. Some substrates utilization degree in the laser treated group was higher than control, such as D-glucosamine, L-glutamine, D-2-Phospho-Glyceric Acid, D-glucosamine-6-phosphate, and D-methionine.

**Conclusion:**

Laser irradiation of *T. rubrum* may lead to changes in the metabolic substrate and metabolic pathway, thus weakening the activity of the strain.

**Supplementary Information:**

The online version contains supplementary material available at 10.1186/s12866-023-02759-3.

## Introduction

*Trichophyton rubrum* (*T. rubrum*) is a dermatophyte responsible for causing the majority of superficial fungal infections worldwide [1]. It can cause infection on the skin, especially the scalp, the inguinal region, the feet, and the nails [1]. These infections can be either chronic or acute with mild to moderate dermatological symptoms. Traditional treatment of *T. rubrum* infection includes both external antifungal creams, such as miconazole nitrate, and oral administration of, such as ketoconazole. Both treatments could cause side effects. Common side effects for oral administration include headaches, taste disturbance, dermatitis, anorexia, vomiting, epigastric pain and diarrhea. Generally, the side effects of topical antifungal agents include periungual erythema and burning at the application site [2].

Laser treatments for fungal infections have attracted attention from both clinicians and scientists in recent years, and the fungicidal effect and mechanism of laser therapy have become hot research topics. Vural and coauthors [3] investigated the mechanism of fungal inhibition by laser treatment and reported that, in addition to nonspecific thermal damage, pigments may be another factor influencing the fungicidal effects due to the cell walls of onychomycosis-causing *Trichophyton* species containing considerable melanin [4, 5]. In a similar study, Zhuo et al. [6] found that high dose of laser irradiation to *T. rubrum* caused an increase of reactive oxygen species (ROS), inhibiting the growth of *T. rubrum*. Subsequently, another study [7] confirmed that photoirradiation therapy stimulated massive ROS production in cells, causing oxidative damage, one of the main mechanisms inhibiting fungal growth.

Previous studies on fungal phenotypes are mostly limited to mycelial morphology, growth rate, drug sensitivity, and biofilm-forming ability but rarely focus on metabolic phenotypes. Phenotypic microarrays (PMs) are a new technology that complement genomics and proteomics, which have long been used to identify microbial strains and determine microbial cell metabolic phenotypes [8, 9]. Zhang et al. [10] analyzed about 2,000 phenotypes of *Streptococcus mutans* mutants using the Biolog PM technology and found that the mutants were more tolerant to various inhibitors of target protein synthesis, DNA synthesis, and cell wall biosynthesis. Additionally, Chen and coauthors [11] explored the differences in biological characteristics between wild-type and mutant strains of *Vibrio cholerae* using this technology. Furthermore, the PM technique was applied with genomics and proteomics to study the phenotypic and functional changes of conidia after gene alterations [12].

In the present study, we used PMs to further analyze the phenotypic changes in *T. rubrum* after laser irradiation. The utilization of four types of nutrition sources, including carbon (the Biolog FF microplate), nitrogen (the Biolog PM3 microplate), phosphorus and surfur (the Biolog PM4 microplate) were analyzed with and without laser irradiation. Carbon, nitrogen, phosphorus and surfur are the most important nutrients required for fungal growth. Our goals are to better understand the effects and mechanism of laser irradiation on *T. rubrum* physiology, and to provide new insights on treatment of *T. rubrum* infections.

## Methods

### The source of *T. rubrum*

A strain of *T. rubrum* was isolated from a patient with onychomycosis who has a good response to laser treatment at Beijing Friendship Hospital (Beijing, China) in December 2017. The strain was identified as *T. rubrum* by sequencing the ITS region from its ribosomal DNA using the primers ITS1 and ITS4 from White et al. [13]. The sequence was deposited in the NCBI Nucleotide database with the access number of OP811248. Clinical trial revealed that the strain is sensitive to laser treatment. The strain is maintained at the Beijing Friendship Hospital and available upon request.

### Laser irradiation

The strain *T. rubrum* was cultured in Sabouraud dextrose agar for 7 days at 25 °C. Fungal suspension was prepared with 1.0 McFarland turbidity. One µL of the culture was inoculated on two sides of a 2% Malt Extract Agar (MEA) plate (Biolog Inc., CA, USA). One side was used for the control group and the other side for the laser treatment. The plates were cultured in an incubator at 25℃, and colony growth was observed daily. When the colony grew to 6 mm in diameter (7 days), the laser side was irradiated using a long-pulsed Nd:YAG 1064 nm laser (Beijing Shiji Guangtong Biotechnology Co., Ltd.) with the following parameters: 3 mm spot size; 1 Hz frequency; 30 ms pulse width; 408 J/cm^2^ laser energy; and each colony was given 200 spots. At this condition, the growth of colony is inhibited, as indicated by destruction of membrane structure and apoptotic cell death [14].

### Metabolic phenotype analysis

Spores of *T. rubrum* were collected by rolling a sterile cotton swab on the surface of the colony. They were then dispersed into the inoculum (0.25% Gellan Gum and 0.03% Tween-40) and the fungal suspension turbidity was determined. The transmittance of 75% for the FF microplate (carbon sources) and 62% for the PM microplates (nitrogen or phosphorus and sulfur source) was used following the instruction of the manufacturer (Biolog Inc). The prepared fungal suspensions were inoculated into FF (95 carbon sources), PM3 (95 nitrogen source), and PM4 (59 phosphorus source and 39 sulfur source) microplates following manufacturer’s instruction (Biolog Inc) and then cultured in an OmniLog phenotype analysis system (Biolog Inc). The degree of utilization was calculated based on the chromogenic reactions. The OmniLog system software measures the color intensity of the reaction wells and changes over time [8]. Each plate contains a negative control to ensure the color intensity was read correctly. The color intensity above the negative control was considered as positive. Triplet experiments were performed from the 3 independently grown colony plates.

### Data processing and statistical analysis

Three independent experiments were performed and data were presented as mean ± standard error. Data analysis for metabolic profiling of *T. rubrum* was conducted using Kinetic and Parametric software from Biolog Inc. D5E_OKA_data.exe was used to collect the color intensity data and OL_FM_1.2.exe was used for data analysis. Phenotypes were determined based on the area under the kinetic curve of dye formation, which is the sum of the color intensities from all time points [8].

A paired *t*-test was conducted to compare the control and the laser treated group, and *P* < 0.05 was considered as statistically significant. The substances metabolized in the control group and laser group were selected, and two independent sample *t*-tests were performed to compare the difference in the degree of metabolism between the two groups. *P* < 0.05 was considered statistically significant.

## Results

### Laser irradiation causes reduction in carbon source utilization

**Fig. 1:**
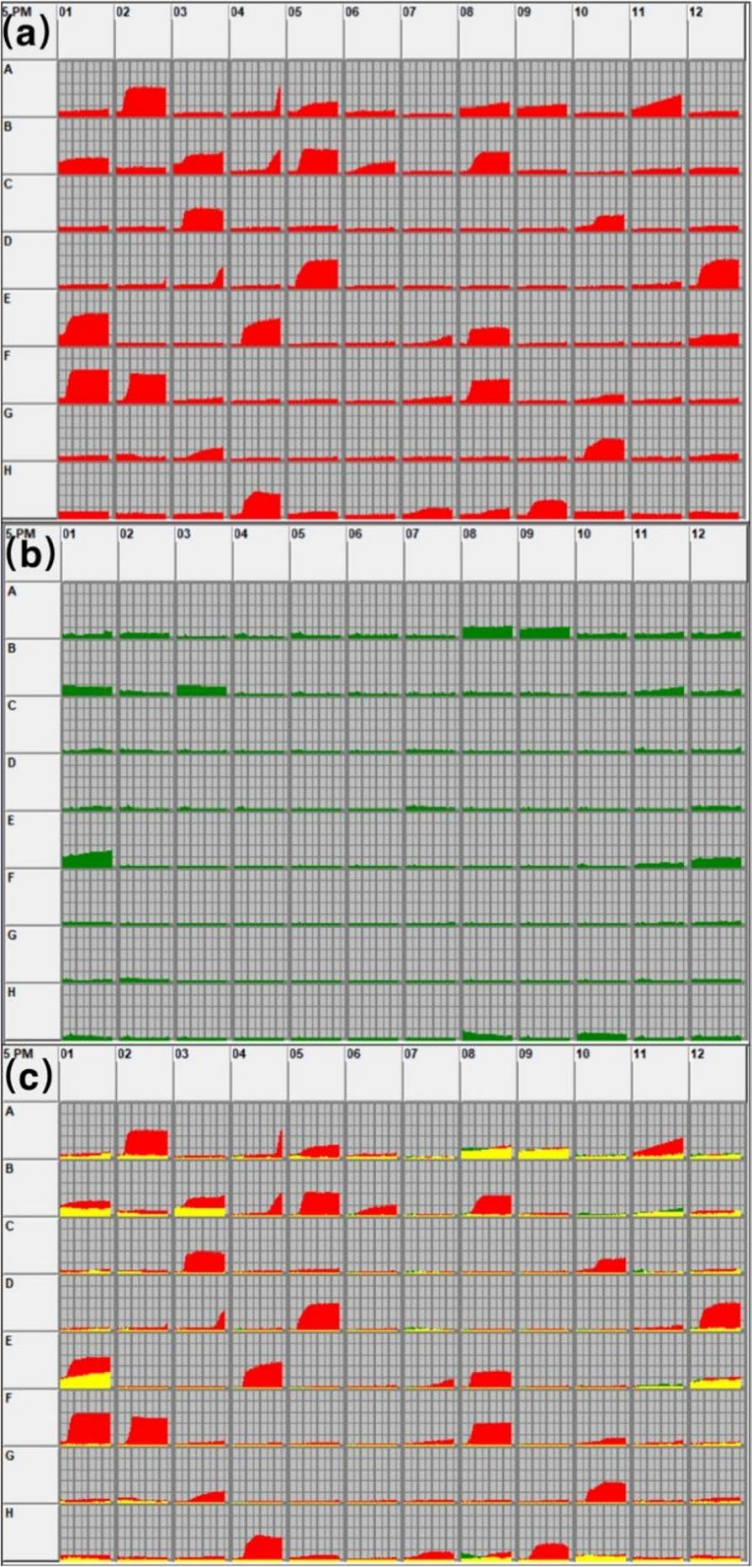
Utilization of carbon source substrates in the control group (**a**) and laser group (**b**), area of carbon source substrate utilization between the two groups (**c**), yellow indicates overlapping substrates between the two groups

**Fig. 2:**
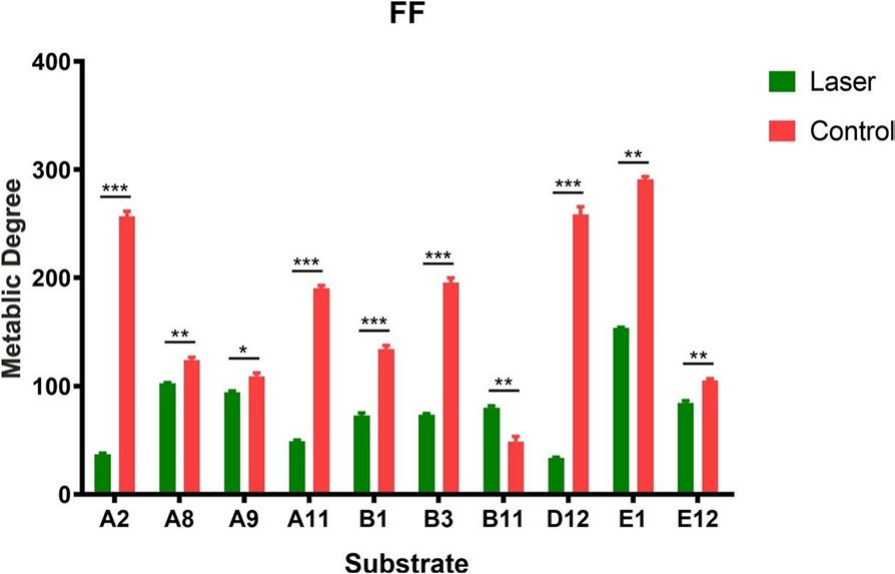
Comparison of the degree of utilization of metabolic carbon source substrates between the two groups at 252 h (red, the control group; green, the laser group) ^*^P < 0.05, ^**^P < 0.01, ^***^P < 0.001. Data were presented as mean ± standard error from three independent experiments. A2 = Tween 80, A8 = D-Arabinose, A9 = L-Arabinose, A11 = Arbutin, B1 = a-Cyclodextrin, B3 = Dextrin, B11 = D-Glucosamine, D12 = L-Rhamnose, E1 = D-Ribose, E12 = D-Xylose

In the FF microplate (carbon source), *T. rubrum* of the control group utilized 33 carbon source substrates, while only 10 for the laser treated group (Table 1). The substance utilization degree was higher in the control group than the laser group, except for D-Glucosamine (B11) (Figs. 1 and 2). These results suggest that laser irradiation causes reduction on carbon source substrate utilization.

**Table 1:** Carbon source utilization between control and laser treated groups

Plate location	Substrate	Control	Laser treated
A2	Tween80	YES	YES
A4	N-Acetyl-ß-D-Glucosamine	YES	
A5	N-Acetyl-ß-D-Mannosamine	YES	
A8	D-Arabinose	YES	YES
A9	L-Arabinose	YES	YES
A11	Arbutin	YES	YES
B1	a-Cyclodextrin	YES	YES
B3	Dextrin	YES	YES
B4	i-Erythritol	YES	
B5	D-Fructose	YES	
B6	L-Fucose	YES	
B8	D-Galacturonic Acid	YES	
B11	D-Glucosamine	YES	YES
C3	D-Glucuronic Acid	YES	
C10	Maltitol	YES	
D3	D-Melezitose	YES	
D5	a-Methyl-D-Galactoside	YES	
D12	L-Rhamnose	YES	YES
E1	D-Ribose	YES	YES
E4	D-Sorbitol	YES	
E7	Sucrose	YES	
E8	D-Tagatose	YES	
E12	D-Xylose	YES	YES
F1	y-Aminobutyric Acid	YES	
F2	Bromosuccinic Acid	YES	
F7	a-Ketoglutaric Acid	YES	
F8	D-Lactic Acid Methyl Ester	YES	
F10	D-Malic Acid	YES	
G3	Succinamic Acid	YES	
G10	L-Asparagine	YES	
H4	L-Proline	YES	
H7	L-Threonine	YES	
H9	Putrescine	YES	

### Laser irradiation causes reduction in nitrogen substrate utilization

**Fig. 3:**
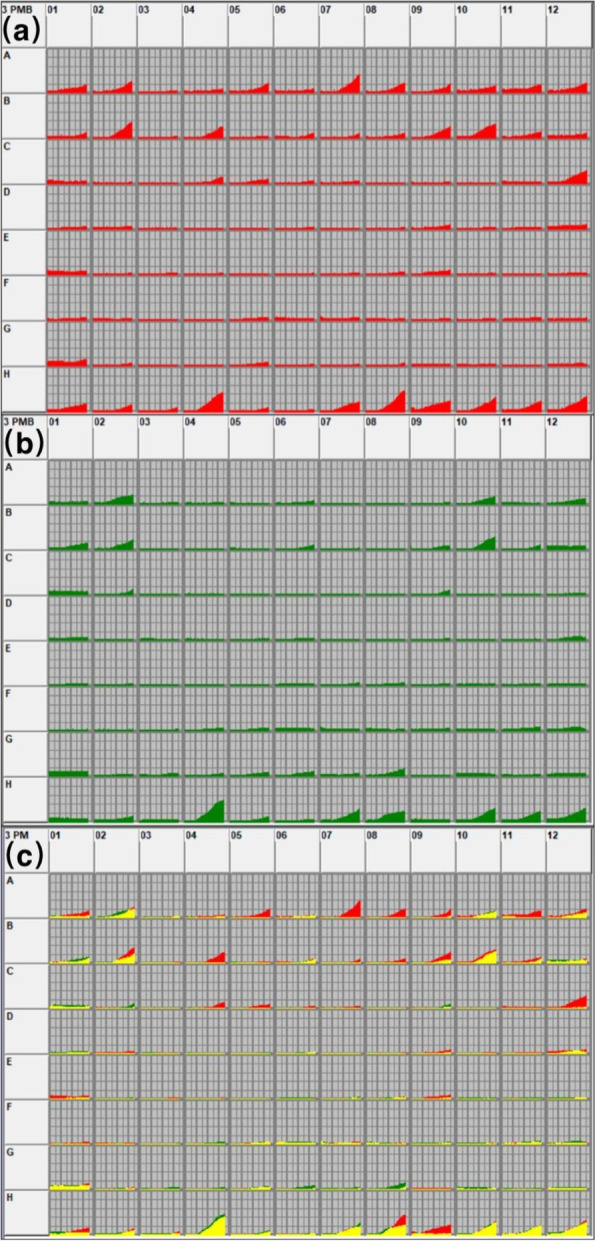
Utilization of nitrogen source substrates in the control group (**a**) and the laser group (**b**). Area of nitrogen source substrate utilization between the two groups (**c**), yellow indicates the overlapping substrates between the two groups

**Fig. 4:**
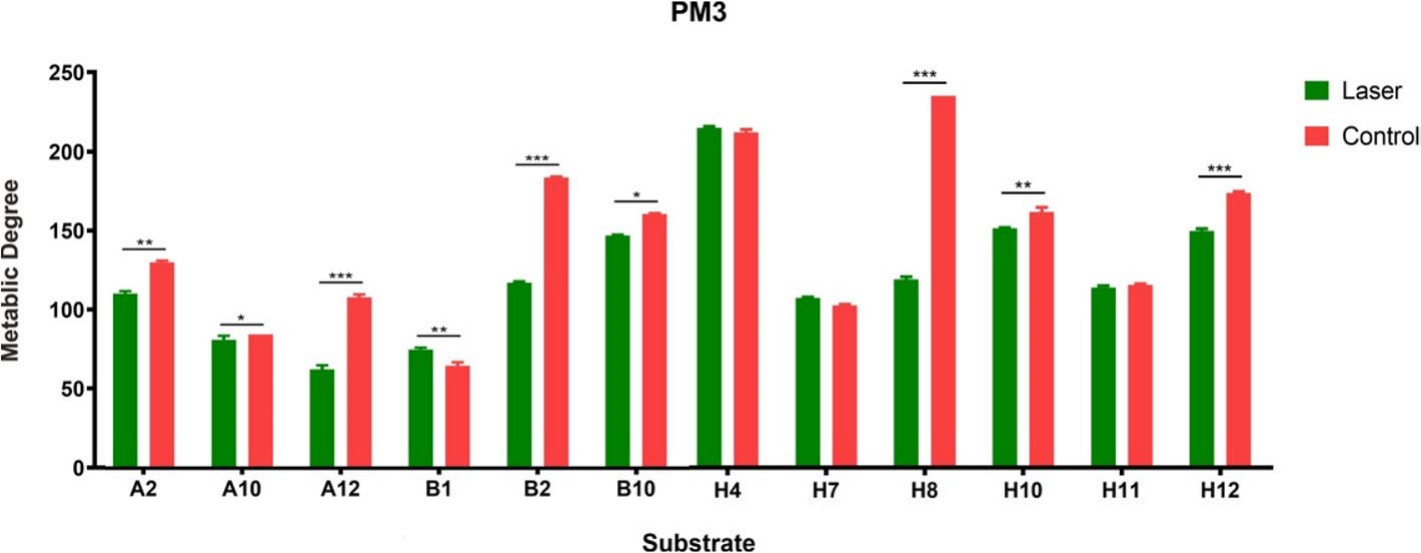
Comparison of the degree of utilization of metabolic nitrogen source substrates between the two groups at 252 h (red, the control group; green, the laser treated group) ^*^P < 0.05, ^**^P < 0.01, ^***^P < 0.001. Data were presented as mean ± standard error from three independent experiments. A2 = Ammonia, A10 = L-Aspartic Acid, A12 = L-Glutamic Acid, B1 = L-Glutamine, B2 = Glycine, B10 = L-Serine, H4 = Ala-Gly, H7 = Ala-Thr, H8 = Gly-Asn, H10 = Gly-Glu, H11 = Gly-Met, H12 = Met-Ala

In the PM3 microplate (nitrogen sources), 20 substrates were utilized by *T. rubrum* of the control group, while only 12 in the laser treated group (Table 2). Among the substrates utilized by both groups, the degree of utilization of majority of the nitrogen substrates (83.3%, 10/12) was lower in the laser treated group. Only the utilization of L-Aspartic Acid (A10) and L-Glutamine (B1) was higher in the laser treated group. The utilization of Ala-Gly (H4), Ala-Thr (H7) and Gly-Met (H11) showed no statistical difference between the two groups (Figs. 3 and 4).

**Table 2:** Nitrogen source utilization between control and laser treated groups

Plate location	Substrate	Control	Laser treated
A2	Ammonia	YES	YES
A7	L-Alanine	YES	
A8	L-Arginine	YES	
A9	L- Asparagine	YES	
A10	L-Aspartic Acid	YES	YES
A12	L-Glutamic Acid	YES	YES
B1	L-Glutamine	YES	YES
B2	Glycine	YES	YES
B4	L-Isoleucine	YES	
B9	L-Proline	YES	
B10	L-Serine	YES	YES
C12	L-Ornithine	YES	
H1	Ala-Asp	YES	
H4	Ala-Gly	YES	YES
H7	Ala-Thr	YES	YES
H8	Gly-Asn	YES	YES
H9	Gly-Gln	YES	
H10	Gly-Glu	YES	YES
H11	Gly-Met	YES	YES
H12	Met-Ala	YES	YES

### Laser irradiation has less effect in sulfur-phosphorus source substrate utilization

**Fig. 5:**
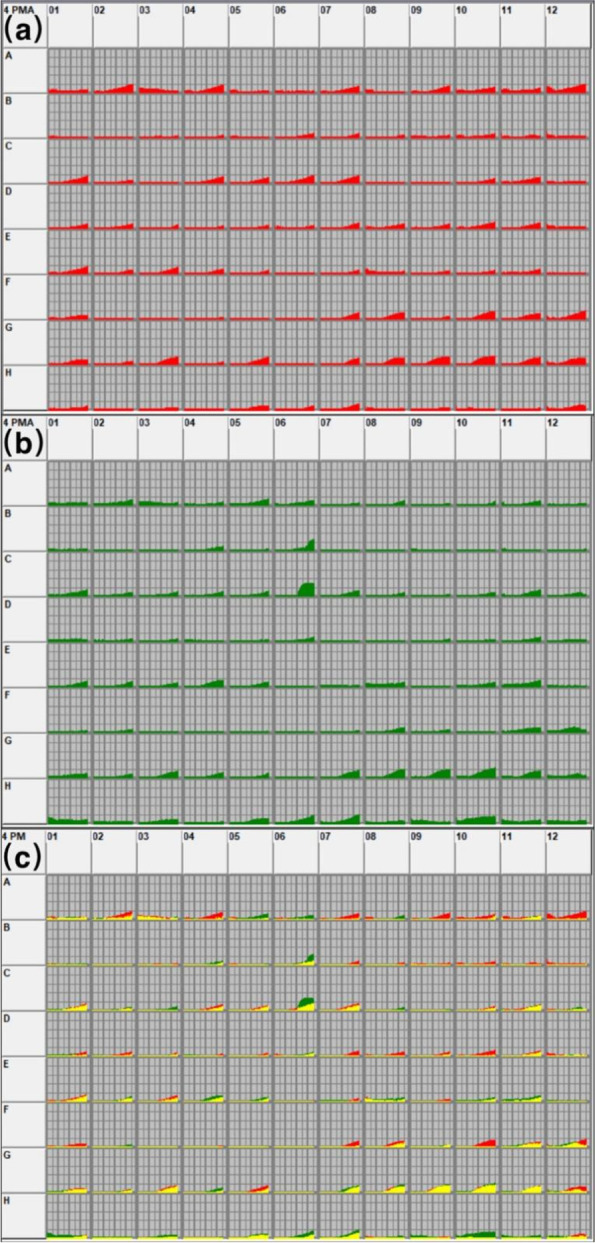
Utilization of sulfur-phosphorus source substrates in the control group (**a**) and the laser treated group (**b**). Area of sulfur-phosphorus source substrate utilization between the two groups (**c**), yellow indicates overlapping substrates between the two groups

**Fig. 6:**
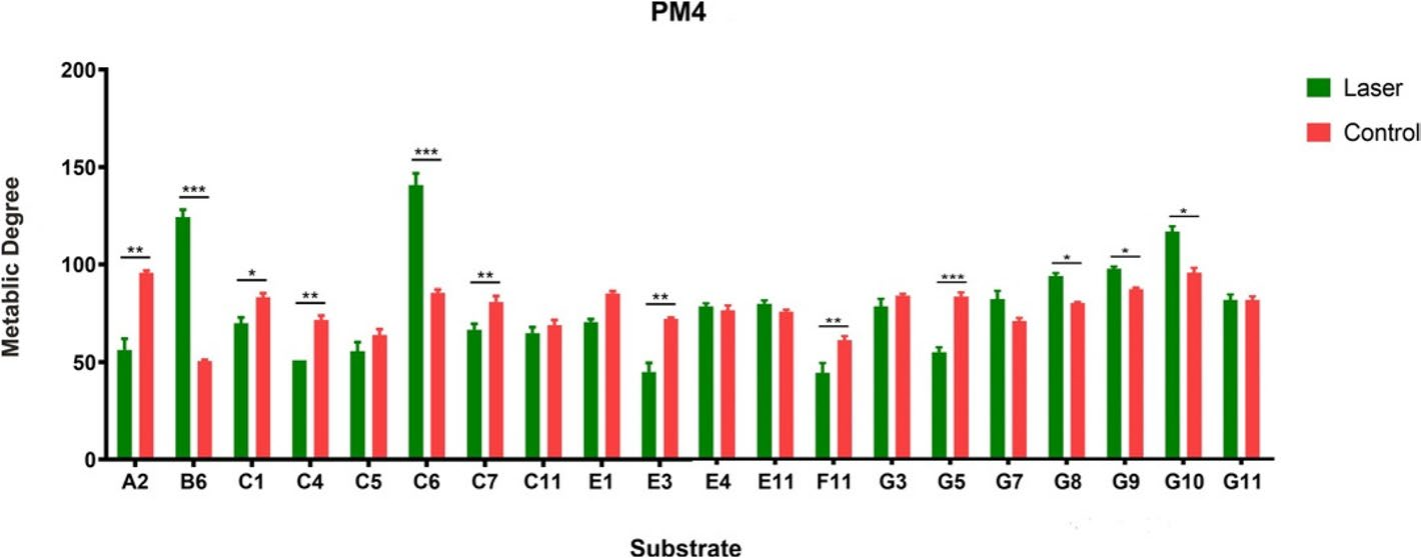
Comparison of the degree of utilization of metabolic sulfur-phosphorus source substrates between the two groups at 252 h (red, control group; green, laser group) ^*^P < 0.05, ^**^P < 0.01, ^***^P < 0.001. Data were presented as mean ± standard error from three independent experiments. A2 = Phosphate, B6 = D-2-Phospho-Glyceric Acid, C1 = Phosphoenol Pyruvate, C4 = D-Glucose-6-Phosphate, C5 = 2-Deoxy-D-Glucose 6-Phosphate, C6 = D-Glucosamine-6-Phosphate, C7 = 6-Phospho-Gluconic Acid, C11 = Cytidine-2’,3’-cyclic monophosphate, E1 = O-Phospho-D-Tyrosine, E3 = Phosphocreatine, E4 = Phosphoryl Choline, E11 = Inositol Hexaphosphate, F11 = Cysteamine, G3 = Cystathionine, G5 = Glutathione, G7 = L-Methionine, G8 = D-Methionine, G9 = Glycyl-L-Methionine, G10 = N-Acetyl-D, L- Methionine, G11 = L-Methionine Sulfoxide

In the PM4 microplate (sulfur-phosphorus substrates), the control group utilized 29 substrates, while the laser treated group only utilized 20 (Table 3). Among the substrates utilized by both groups, almost half (8/20) showed no statistical difference between the two groups for their degree of sulfur-phosphorus substrate utilization. The utilization of D-2-Phospho-Glyceric Acid (B6), D-Glucosamine-6-Phosphate (C6), D-Methionine (G8), Glycyl-L-Methionine (G9) and N-Acety l-D, L-Methionine (G10) was higher in the laser treated group (Figs. 5 and 6).

**Table 3:** Sulfur-phosphorus source utilization between control and laser treated groups

Plate location	Substrate	Control	Laser treated
A2	Phosphate	YES	YES
A4	Trimeta-phosphate	YES	
A7	Hypophosphite	YES	
A9	Adenosine-3’-monophosphate	YES	
A12	Adenosine-3’,5’-cyclicmonophosphate	YES	
B6	D-2-Phospho-Glyceric Acid	YES	YES
C1	Phosphoenol Pyruvate	YES	YES
C4	D-Glucose-6-Phosphate	YES	YES
C5	2-Deoxy-D-Glucose 6-Phosphate	YES	YES
C6	D-Glucosamine-6-Phosphate	YES	YES
C7	6-Phospho-Gluconic Acid	YES	YES
C11	Cytidine-2’,3’-cyclicmonophosphate	YES	YES
E1	O-Phospho-D-Tyrosine	YES	YES
E3	Phosphocreatine	YES	YES
E4	Phosphoryl Choline	ES	YES
E11	Inositol Hexaphosphate	YES	YES
F7	L-Cysteine	YES	
F8	D-Cysteine	YES	
F10	L-Cysteic Acid	YES	
F11	Cysteamine	YES	YES
F12	L-Cysteine Sulfinic Acid	YES	
G3	Cystathionine	YES	YES
G5	Glutathione	YES	YES
G7	L-Methionine	YES	YES
G8	D-Methionine	YES	YES
G9	Glycyl-L-Methionine	YES	YES
G10	N-Acetyl-D,L-Methionine	YES	YES
G11	L-Methionine Sulfoxide	YES	YES
G12	L-Methionine Sulfone	YES	

## Discussion

Traditionally, *T. rubrum* phenotypes were mostly detected using carbon source assimilation tests, during which the types of tested substances are limited, the operation is complicated, and only one substance can be tested at once. PMs overcome this defect by detecting the color response during the respiratory metabolism of living cells, enabling the collection of large amounts of data on the microbial utilization of various nutrients. In the present study, we analyzed the metabolic differences between the laser treated and non-treated (control) *T. rubrum* groups. First, compared with laser-irradiated *T. rubrum*, the control group efficiently utilized saccharides (e.g., arabinose, D-fructose, sucrose) and organic acids (e.g., glucuronic acid, D-malic acid, and aminobutyric acid) as carbon sources as well as some amino acids (e.g., alanine, arginine, serine) as nitrogen sources for growth. Many sulfur and phosphorus source substrates were also utilized, but their utilization degree was lower than that of the carbon and nitrogen source substrates. Second, compared with control *T. rubrum*, the number of substrates utilized by *T. rubrum* after laser irradiation were significantly reduced, including saccharides, organic acids, and a variety of amino acids, indicating that the process of energy generation may be damaged by laser [15]. Third, the substrate utilization degree in the laser treated group was higher than that in the control group for several substances, such as carbon source substrate D-glucosamine, nitrogen source substrates L-aspartic acid and L-glutamine, phosphorus source substrate D-glucosamine-6-phosphate, and sulfur source substrate D-methionine. This could be related to a self-protection mechanism or the fact that laser irradiation promotes *T. rubrum* apoptosis. Further studies could help us better understand whether this is self-protection or cell death, and the associated molecular pathway.

Results from the carbon source test showed that the types of metabolized substrates were similar between the two groups, but the number of metabolized substrates was different. The laser treated group only used 30% of the substrates used by the control group (Figs. 1 and 2). Saccharides are the most abundant carbon source and may play a key role in the survival of *T. rubrum*. The tricarboxylic acid cycle (TCA) is not only a common metabolic pathway in aerobic organisms but also the final metabolic pathway of three major nutrients (saccharides, lipids, and amino acids). After the laser irradiation of *T. rubrum*, a variety of metabolites involved in the TCA were inhibited, including alpha-ketoglutarate, D-malic acid, succinic acid, L-asparagine, L-proline, L-threonine, and putrescine. Therefore, we speculate that after laser irradiation, some substrates cannot be utilized by *T. rubrum* due to the damages of genes that modulate the TCA, reducing the metabolic capacity of *T. rubrum*. In addition, laser irradiation reduced the metabolic capacity of *T. rubrum* for some substrates involved in glycolysis, such as D-glucuronic acid and maltitol. On this basis, it is speculated that laser irradiation may destroy multiple key genes involved in glycolysis or the TCA of *T. rubrum*, thus affecting the metabolism of corresponding substrates. Although the TCA is the main glucose metabolism pathway, it is not the only one. When the TCA is inhibited, the pentose phosphate pathway can be used to metabolize carbohydrates [16]. In this study, *T. rubrum* irradiated by laser could still utilize some carbon source substrates in the pentose phosphate pathway, including D-arabinose, L-arabinose, D-ribose, and D-xylose.

D-glucosamine exists widely in the chitin and glycoproteins of bacterial cell walls and the chitin of fungal cell walls in the form of N-acetylglucosamine [17]. The transport of glucosamine to cells through glucose transporters [18] will directly increase the flux through the hexosamine biosynthesis pathway, enhancing O-linked β-N-acetylglucosamine (O-GlcNAc) glycosylation [19]. The O-GlcNAc protein is an endogenous protective mechanism triggered by stress and is considered an “emergency receptor” [20]. Increases in O-GlcNAc levels have been shown to contribute to higher heat resistance in cells [20]. In our study, we showed that the utilization of D-glucosamine is increased after laser irradiation. We suspect that laser irradiation of *T. rubrum* triggers this self-protection mechanism, resulting in a higher degree of D-glucosamine utilization.

Microbial growth and product synthesis require nitrogen sources, which are mainly used for the biosynthesis of amino acids, proteins, nucleic acids, and nitrogen metabolites. Research on the sporulation of *T. rubrum* under different nitrogen sources has shown that *T. rubrum* can grow normally without a carbon source when nitrogen sources are available [21], suggesting that nitrogen substrates are vital to *T. rubrum*. In our PM3 microplate with nitrogen sources, the control group effectively utilized 20 nitrogen-containing substances, mainly amino acids involved in glucose and nucleic acid metabolism and a few dipeptides. However, only 12 nitrogen substrates were utilized by *T. rubrum* after laser irradiation, and the degree of metabolism of most substrates was significantly lower than that of the control group.

Amino acids are precursors for purine and pyrimidine biosynthesis. Amino acid metabolism participates in the TCA and is the central point of glucose, lipid, and amino acid metabolism. After laser irradiation, *T. rubrum* lost the ability to utilize alanine, arginine, asparaginate, isoleucine, proline and ornithine, and its degree of utilization of glutamate, glycine, and serine decreased, indicating that amino acid metabolism was inhibited. Moreover, laser irradiation increased the utilization of L-aspartic acid and L-glutamine by *T. rubrum*, which may be due to the key roles of glutamine and aspartic acid in cell growth and proliferation. Glutamine and aspartic acid, as intermediate metabolites of glucose metabolism, can participate in the TCA and provide energy for cells [22]. They also provide nitrogen sources for nucleotides, proteins, and other biological macromolecules [23]. The efficient utilization of glutamine and aspartic acid can also affect DNA repair and replication [24] to maintain the survival of bacteria.

Phosphorus absorption and utilization play important roles in biological processes, such as heredity, energy metabolism, cell membrane integrity, and intracellular signal transduction. Organisms have formed a complex phosphate system to regulate phosphorus metabolism [25]. Our analysis showed that without laser irradiation, *T. rubrum* effectively utilized phosphate, trimetaphosphate, hypophosphate, adenosine-3’-monophosphate, adenosine-2’,5’-cyclic monophosphate, D-2-phosphoglyceric acid, D-glucose-6-phosphoric acid, 2-deoxy-D-glucose-6-phosphoric acid, 6-phosphoric acid-gluconic acid, creatine phosphate, and choline phosphate, which are involved in gluconeogenesis, phospholipid metabolism, nucleotide metabolism, energy transport, and signal transduction. After laser irradiation, the ability of *T. rubrum* to utilize these phosphorous substances was lost or significantly reduced.

**Fig. 7:**
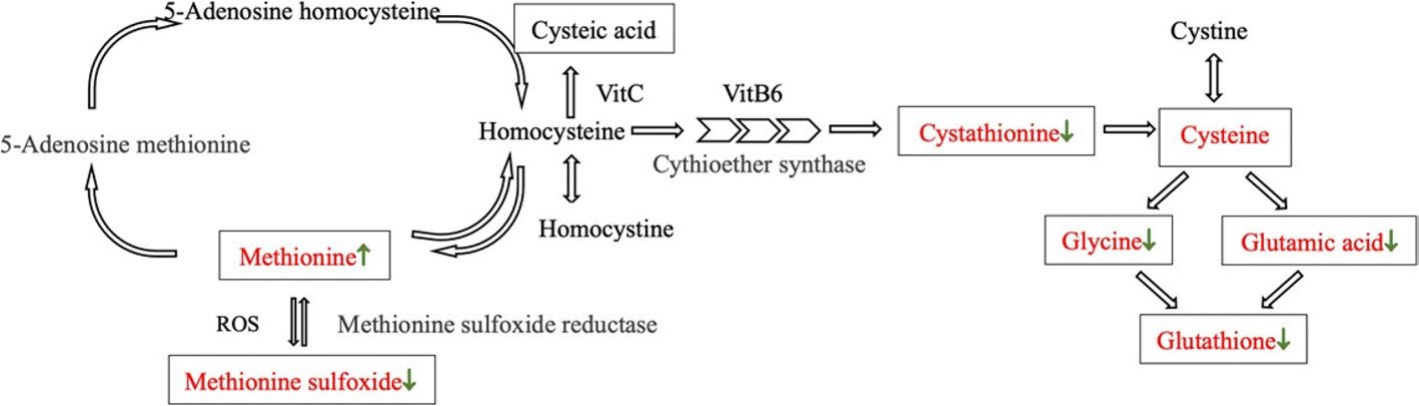
Methionine and glutathione synthesis pathways. Hollow black arrows indicate the direction of reaction; Solid arrow (↑) indicates utilization in the laser treated group is increased, while (↓) indicates utilization in the laser treated group is decreased compared with the control

Sulfur-containing amino acids include methionine, cysteine, and cystine. Methionine is one of the most easily oxidized amino acids in organisms and the activity center of proteins. Methionine in proteins can function normally only if the correct structure is maintained. However, under oxidative stress, methionine is very likely to be oxidized to methionine sulfoxide [26]. The increase in methionine utilization capability in the laser treated group may be related to increased ROS levels after laser irradiation [6, 27]. ROS can oxidize methionine to methionine sulfoxide, which may affect a variety of biological functions [28]. Methionine sulfoxide reductase is widely distributed in pathogens and can reduce methionine sulfoxide to methionine. Under normal conditions, methionine sulfoxide reductase can reverse the above-mentioned oxidation reaction, protecting against oxidative stress [29]. However, after laser irradiation, the capability of *T. rubrum* to utilize methionine sulfoxide decreased, and this oxidation could not be prevented or reversed (Fig. 7).

Glutathione is a low-molecular-mass polypeptide composed of glycine, cysteine, and glutamic acid. Glutathione can remove superoxide ions and other free radicals to prevent damage to the cell [30]. Studies have shown that the laser irradiation of *T. rubrum* leads to an increase in ROS [6, 31]. We expect *T. rubrum* in the laser treated group to utilize more glutathione to resist the damage caused by superoxidation. However, our results revealed that the degree of glutathione utilization in the laser group was lower than that in the control group, which may be because glutathione is affected by multiple metabolic enzymes. The laser irradiation of *T. rubrum* could lead to glutathione synthesis dysfunction, limiting the ability to maintain the reduction under oxidative stress and reducing the glutathione utilization capacity of the laser group.

## Conclusion

In conclusion, this study demonstrated that the overall metabolic capacity of *T. rubrum* decreased after laser irradiation, but not all substances were inhibited. Therefore, we believe that the laser irradiation parameters used in our experiment have a fungistatic effect, rather than a fungicidal effect, consistent with the results of our previous work. Our study provides new insights on the fungistatic and fungicidal effects of laser treatment. The limitation of this study lies in that we could only measure the degree of metabolic capacity of *T. rubrum* irradiated by laser. More experimental studies are needed to explore the specific mechanisms underlying these changes to confirm the effect of laser treatment on the metabolic phenotypes of *T. rubrum*.

## Supplementary Information

**Additional file 1.**

**Additional file 2.**

## Data Availability

The data during the current study are available in the national center for biotechnology information (NCBI) under accessions number OP811248 (https://www.ncbi.nlm.nih.gov/nuccore/OP811248). Other data generated or analyzed during this study are included in the published article and its supplementary.
