# Supplementary material for: Metabolic phenotype analysis of Trichophyton rubrum after laser irradiation
Source: BMC Microbiol. 2023 Jan 21;23:24. doi: 10.1186/s12866-023-02759-3 (PMC9862980; doi:10.1186/s12866-023-02759-3)
Supplement: Supplementary file 1 — Additional file 1. [file 12866_2023_2759_MOESM1_ESM.pdf]

FF microplate-Carbon sources substrates

A1<br>Water	A2<br>Tween 80	A3<br>N-Acetyl-D-Galactosamine	A4<br>N-Acetyl-ß-D-Glucosamine	A5<br>N-Acetyl-ß-D-Mannosamine	A6<br>Adonitol	A7<br>Amygdalin	A8<br>D-Arabinose	A9<br>L-Arabinose	A10<br>D-Arabitol	A11<br>Arbutin	A12<br>D-Cellobiose
B1<br>a-Cyclodextrin	B2<br>ß-Cyclodextrin	B3<br>Dextrin	B4<br>i-Erythritol	B5<br>D-Fructose	B6<br>L-Fucose	B7<br>D-Galactose	B8<br>D-Galacturonic Acid	B9<br>Gentiobiose	B10<br>D-Gluconic Acid	B11<br>D-Glucosamine	B12<br>a-D-Glucose
C1<br>a-D-Glucose-1<br>-Phosphate	C2<br>Glucuronamide	C3<br>D-Glucuronic Acid	C4<br>Glycerol	C5<br>Glycogen	C6<br>m-Inositol	C7<br>2-Keto-D-Gluconic Acid	C8<br>a-D-Lactose	C9<br>Lactulose	C10<br>Maltitol	C11<br>Maltose	C12<br>Maltotriose
D1<br>D-Mannitol	D2<br>D-Mannose	D3<br>D-Melezitose	D4<br>D-Melibiose	D5<br>a-Methyl-D-Galactoside	D6<br>ß-Methyl-D-Galactoside	D7<br>a-Methyl-D-Glucoside	D8<br>ß-Methyl-D-Glucoside	D9<br>Palatinose	D10<br>D-Psicose	D11<br>D-Raffinose	D12<br>L-Rhamnose
E1<br>D-Ribose	E2<br>Salicin	E3<br>Sedoheptulosan	E4<br>D-Sorbitol	E5<br>L-Sorbose	E6<br>Stachyose	E7<br>Sucrose	E8<br>D-Tagatose	E9<br>D-Trehalose	E10<br>Turanose	E11<br>Xylitol	E12<br>D-Xylose
F1<br>y-Aminobutyric Acid	F2<br>Bromosuccinic Acid	F3<br>Fumaric Acid	F4<br>ß-Hydroxybutyric Acid	F5<br>y-Hydroxybutyric Acid	F6<br>p-Hydroxy-phenylacetic Acid	F7<br>a-Ketoglutaric Acid	F8<br>D-Lactic Acid Methyl Ester	F9<br>L-Lactic Acid	F10<br>D-Malic Acid	F11<br>L-Malic Acid	F12<br>Quinic Acid
G1<br>D-Saccharic Acid	G2<br>Sebacic Acid	G3<br>Succinamic Acid	G4<br>Succinic Acid	G5<br>Succinic Acid Mono-Methyl Ester	G6<br>N-Acetyl-L-Glutamic Acid	G7<br>L-Alaninamide	G8<br>L-Alanine	G9<br>L-Alanyl-Glycine	G10<br>L-Asparagine	G11<br>L-Aspartic Acid	G12<br>L-Glutamic Acid
H1<br>Gycyl-L-Glutamic Acid	H2<br>L-Ornithine	H3<br>L-Phenylalanine	H4<br>L-Proline	H5<br>L-Pyroglutamic Acid	H6<br>L-Serine	H7<br>L-Threonine	H8<br>2-Aminoethanol	H9<br>Putrescine	H10<br>Adenosine	H11<br>Uridine	H12<br>Adenosine-5'-Monophosphate

PM 3 microplate-Nitrogen source substrates

A1<br>Negative Control	A2<br>Ammonia	A3<br>Nitrite	A4<br>Nitrate	A5<br>Urea	A6<br>Biuret	A7<br>L - Alanine	A8<br>L - Arginine	A9<br>L - Asparagine	A10<br>L - Aspartic Acid	A11<br>L - Cysteine	A12<br>L - GlutamicAcid
B1<br>L - Glutamine	B2<br>Glycine	B3<br>L - Histidine	B4<br>L - Isoleucine	B5<br>L - Leucine	B6<br>L - Lysine	B7<br>L - Methionine	B8<br>L - Phenylalanine	B9<br>L - Proline	B10<br>L - Serine	B11<br>L -Threonine	B12<br>L -Tryptophan
C1<br>L -Tyrosine	C2<br>L - Valine	C3<br>D - Alanine	C4<br>D - Asparagine	C5<br>D - Aspartic Acid	C6<br>D - Glutamic Acid	C7<br>D - Lysine	C8<br>D - Serine	C9<br>D - Valine	C10<br>L - Citrulline	C11<br>L - Homoserine	C12<br>L - Ornithine
D - 1<br>N - Acetyl - D ,L - Glutamic Acid	D2<br>N - Phthaloyl - L - Glutamic Acid	D3<br>L - Pyroglutamic Acid	D4<br>Hydroxylamine	D5<br>Methylamine	D6<br>N - Amylamine	D7<br>N - Butylamine	D8<br>Ethylamine	D9<br>Ethanolamine	D10<br>Ethylenediamine	D11<br>Putrescine	D12<br>Agmatine
E1<br>Histamine	E2<br>β - Phenylethyl - amine	E3<br>Tyramine	E4<br>Acetamide	E5<br>Formamide	E6<br>Glucuronamide	E7<br>D, L - Lactamide	E8<br>D - Glucosamine	E9<br>D - Galactosamine	E10<br>D - Mannosamine	E11<br>N - Acetyl - D - Glucosamine	E12<br>N - Acetyl - D - Galactosamine
F1<br>N - Acetyl - D - Mannosamine	F2<br>Adenine	F3<br>Adenosine	F4<br>Cytidine	F5<br>Cytosine	F6<br>Guanine	F7<br>Guanosine	F8<br>Thymine	F9<br>Thymidine	F10<br>Uracil	F11<br>Uridine	F12<br>Inosine
G1<br>Xanthine	G2<br>Xanthosine	G3<br>UricAcid	G4<br>Alloxan	G5<br>Allantoin	G6<br>Parabanic Acid	G7<br>D, L - α -A mino - N - Butyric Acid	G8<br>γ - Amino - N - Butyric Acid	G9<br>ε -Amino - N - Caproic Acid	G1 0<br>D, L - α -Ami o - - Caprylic Acid	G1 1<br>δ -Amino - N - Valeric Acid	G1 2<br>α -Amino - N - Valeric Acid
H1<br>Ala - Asp	H2<br>Ala - Gln	H3<br>Ala - Glu	H4<br>Ala - Gly	H5<br>Ala - His	H6<br>Ala - Leu	H7<br>Ala -Thr	H8<br>Gly - Asn	H9<br>Gly - Gln	H10<br>Gly - Glu	H11<br>Gly - Met	H12<br>Met -Ala

PM4 Microplate- Phosphorus and sulfur source substrates

A1<br>Negative Control	A2<br>Phosphate	A3<br>Pyrophosphate	A4<br>Trimeta - phosphate	A5<br>Tripoly - phosphate	A6<br>Triethyl Phosphate	A7<br>Hypophosphite	A8<br>Adenosine - 2’ - monophosphate	A9<br>Adenosine-3’- monophosphate	A10<br>Adenosine - 5’ - monophosphate	A11<br>Adenosine - 2’, 3’ - cyclic monophosphate	A12<br>Adenosine- 3’, 5’-cycl ic monophosphate
B1<br>Thiophosphate	B2<br>Dithiophosphate	B3<br>D, L - α - Glycerol Phosphate	B4<br>β - Glycerol Phosphate	B5<br>Carbamyl Phosphate	B6<br>D - 2 - Phospho - Glyceric Acid	B7<br>D - 3 - Phospho - Glyceric Acid	B8<br>Guanosine - 2’ - monophosphate	B9<br>Guanosine - 3’ - monophosphate	B10<br>Guanosine - 5’ - monophospha te	B11<br>Guanosine - 2’, 3’ - cyclic monophosphate	B12<br>Guanosine - 3’, 5’ - cyclic monophosphate
C1<br>Phosphoenol Pyruvate	C2<br>Phospho - Glycolic Acid	C3<br>D - Glucose - 1 - Phosphate	C4<br>D - Glucose - 6 - Phosphate	C5<br>2 - Deoxy - D - Glucose 6 - Phosphate	C6<br>D - Gl ucosamine - 6 - Phosphate	C7<br>6 - Phospho - Gluconic Acid	C8<br>Cytidine - 2’ - monophosphate	C9<br>Cytidine - 3’ - monophosphate	C10<br>Cytidine - 5’ - monophosphate	C11<br>Cytidine - 2’ ,3’ - cyclic monophosphate	C12<br>Cytidine - 3’ ,5’ - cyclic monophosphate
D1<br>D - Mannose - 1 - Phosphate	D2<br>D - Mannose - 6 - Phosphate	D3<br>Cysteamine - S - Phosphate	D4<br>Phospho - L - Arginine	D5<br>O - Phospho - D - Serine	D6<br>O - Phospho - L - Serine	D7<br>O - Phospho - L - Threonine	D8<br>Uridine - 2’ - monophosphate	D9<br>Uridine - 3’ - monophosphate	D10<br>Uridine - 5’ - monophosphate	D11<br>Uridine - 2’ ,3’ - cyclic monophosphate	D12<br>Uridine - 3’ ,5’ - cyclic monophosphate
E1<br>O - Phospho - D - Tyrosine	E2<br>O - Phospho - L - Tyrosine	E3<br>Phosphocreatine	E4<br>Phosphoryl Choline	E5<br>O - Phosphoryl - Ethanolamine	E6<br>Phosphono Aceti Acid	E7<br>2 - A minoethyl Phosphonic Acid	E8<br>Methylene Diphosphonic Acid	E9<br>Thymidine - 3 ’ - monophosphate	E10<br>Thymidine - 5 ’ - monophosphate	E11<br>Inositol Hexaphosphate	E12<br>Thymidine 3’, 5’ - cyclic monophosphate
F1<br>Negative Control	F2<br>Sulfate	F3<br>Thiosulfate	F4<br>Tetrathionate	F5<br>Thiophosphate	F6<br>Dithiophosphate	F7<br>L - Cysteine	F8<br>D - Cysteine	F9<br>L - Cysteinyl - Glycine	F10<br>L - Cysteic Acid	F11<br>Cysteamine	F12<br>L - Cysteine Sulfinic Acid
G1<br>N - Acety l - L - Cysteine	G2<br>S - Methyl - L - Cysteine	G3<br>Cystathionine	G4<br>Lanthionine	G5<br>Glutathione	G6<br>D, L - Ethionine	G7<br>L - Methionine	G8<br>D - Methionine	G9<br>Glycyl - L - Methionine	G1 0<br>N - Acetyl - D ,L - Methionine	G1 1<br>L - Methionine Sulfoxide	G1 2<br>L - Methionine Sulfone
H1<br>L - Djenkolic Acid	H2<br>Thiourea	H3<br>1 -Thio - β - D - Glucose	H4<br>D, L - Lipoamide	H5<br>Taurocholic Acid	H6<br>Taurine	H7<br>Hypotaurine	H8<br>p- Amino Benzene Sulfonic Acid	H9<br>Butane Sulfonic Acid	H10<br>2 - Hydroxyethane Sulfonic Acid	H11<br>Methane Sulfonic Acid	H12<br>Tetramethylene Sulfone
