# Supplementary material for: Metabolic phenotype analysis of Trichophyton rubrum after laser irradiation
Source: BMC Microbiol. 2023 Jan 21;23:24. doi: 10.1186/s12866-023-02759-3 (PMC9862980; doi:10.1186/s12866-023-02759-3)
Supplement: Supplementary file 2 — Additional file 2. [file 12866_2023_2759_MOESM2_ESM.docx]

**S3424-ITS ML 666bp**

TGCGGAAGGATCATTAACGCGCAGGCCGGAGGCTGGCCCCCCACGATAGGGACCGACGTTCCATCAGGGGTGAGCAGACGTGCGCCGGCCGTACGCCCCCATTCTTGTCTACCTCACCCGGTTGCCTCGGCGGGCCGCGCTCCCCCTGCCAGGGAGAGCCGTCCGGCGGGCCCCTTCTGGGAGCCTCGAGCCGGACCGCGCCCGCCGGAGGACAGACACCAAGAAAAAATTCTCTGAAGAGCTGTCAGTCTGAGCGTTTAGCAAGCACAATCAGTTAAAACTTTCAACAACGGATCTCTTGGTTCCGGCATCGATGAAGAACGCAGCGAAATGCGATAAGTAATGTGAATTGCAGAATTCCGTGAATCATCGAATCTTTGAACGCACATTGCGCCCTCTGGCATTCCGGGGGGCATGCCTGTTCGAGCGTCATTTCAACCCCTCAAGCCCGGCTTGTGTGATGGACGACCGTCCGGCCCCTCCCTTCGGGGGCGGGACGCGCCCGAAAAGCAGTGGCCAGGCCGCGATTCCGGCTTCCTAGGCGAATGGGCAGCCAATTCAGCGCCCTCAGGACCGGCCGCCCTGGCCCCAATCTTTATATATATATATATCTTTTCAGGTTGACCTCGGATCAGGTAGGGATACCCGCTGAACTTAAGCATATCA

The ITS sequence for Trichophyton rubrum strain in our study is with 100% similarity with the ITS sequences of Trichophyton rubrum strains IHEM: 21696(Accession No.OW986422.1) and IHEM:20850 (Accession No. OW986312.1).So the strain was identified as *Trichophyton* and presumed to be *Trichophyton rubrum.*
